# Analysis of the ocular surface functional unit in episodic migraine

**DOI:** 10.1007/s00417-023-06324-6

**Published:** 2023-12-01

**Authors:** Ágnes Patzkó, Adrienne Csutak, Noémi Tóth, Zsófia Kölkedi, Zoltán Pfund, Gréta Kis-Jakab, Edit Bosnyák, Renáta Rozgonyi, Eszter Szalai

**Affiliations:** 1 Department of Ophthalmology, University of Pécs Medical School, Rákóczi u. 2, 7623 Pécs, Hungary; 2 Department of Neurology, University of Pécs Medical School, Rét u. 2, 7623 Pécs, Hungary

**Keywords:** Cornea, Corneal nerves, Migraine, Dendritic cell, In vivo corneal confocal microscopy

## Abstract

**Aim:**

Migraine is a chronic neurovascular disease that affects the trigeminovascular system. The purpose of this study was to evaluate corneal subbasal nerve fibers, dendritic cells and to measure tear film parameters in migraine.

**Patients and methods:**

87 eyes of 44 patients suffering from migraine with a mean age of 33.23 ± 11.41 years were included in our study. 25 age-matched controls (mean age of 30.16 ± 12.59 years; *P* = 0.162) were recruited. The corneal subbasal plexus and the dendritic cells (DC) were analyzed using in vivo confocal microscopy (Heidelberg Retina Tomograph II Rostock Cornea Module; Heidelberg Engineering GmbH), and the tear film was imaged using LacryDiag (Quantel Medical, France).

**Results:**

Regarding the subbasal nerve fibers of the cornea, none of the examined parameters differed significantly in migraine patients from controls. We found a significant increase in the corneal DC density (*P* < 0.0001) and DC area (*P* < 0.0001) in migraine patients compared to healthy volunteers. DC density showed a positive correlation with the monthly attack frequency (r = 0.32, *P* = 0.041) and the DC area a negative correlation with corneal nerve branch density (r = -0.233, *P* = 0.039), nerve fiber length (r = -0.232, *P* = 0.04) and total branch density (r = -0.233, *P* = 0.039). Using LacryDiag a significant loss of Meibomian gland area could be detected on the superior eyelid (*P* = 0.005) in migraine.

**Conclusions:**

Our results suggest the presence of neuroinflammation in the cornea of migraine patients affecting the peripheral trigeminal system. Dendritic cells surrounding the subbasal plexus may be involved in the activation and modulation of pain in migraine.


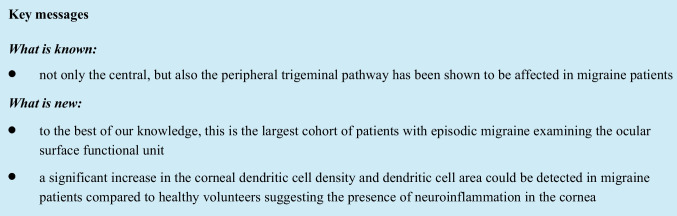


## Introduction

Ocular surface as a functional unit (composed by the cornea, bulbar and tarsal conjunctiva) is covered by three layers of the continuously renewing precorneal tear film [1]. The production of the tear film is controlled by parasympathetic as well as by the sympathetic nervous system. The trigeminal nerve is the primary pathway mediating the parasympathetic regulation of the tear film homeostasis.

Migraine is a common disabling primary headache disorder presenting with a throbbing pain or a pulsing sensation, usually on one side of the head. Commonly associated symptoms are nausea, vomiting, photophobia, and phonophobia [2]. Approximately 16% of the worldwide population is affected by migraine headache, and nearly 30% of these patients experience neurological symptoms related to a transient cortical malfunction, generally known as aura [3]. According to the Global Burden of Disease Study, migraine is the second most prevalent neurological disorder worldwide and is responsible for more disability than all other neurological disorders combined [4–6].

The headache phase of a migraine attack is believed to be triggered by the activation of the nociceptors of the meninges and [7] the intracranial vessels. The nociceptors have their origin in the trigeminal ganglion and get to the dura mater primarily through the ophthalmic branch of the trigeminal nerve (V1) [8]. Although, the pathophysiology of migraine has not been fully elucidated yet, the trigeminovascular theory, describing the recurrent sensitization and activation of the trigeminovascular pathway is widely accepted as having a fundamental role in this highly complex neurological disorder [9, 10]. Recent functional magnetic resonance imaging (MRI) studies confirm the activation of the peripheral and central trigeminal system in migraine [11]. The recurrent activation of the trigeminovascular pathway leads to consequential structural and functional changes in the central nervous system of genetically susceptible individuals [10]. There is also growing evidence on abnormalities affecting the peripheral trigeminal afferent nerves in humans in vivo [12]. The trigeminal nerve supplies the corneal subbasal nerve system, which is mainly responsible for the sensation of touch, pain and tear film characteristics. In vivo confocal microscopy (IVCM) is a noninvasive imaging and diagnostic tool in the investigation of the corneal microstructure that enables a high resolution evaluation and quantitative analysis of the ocular surface at the cellular level [13]. Previous IVCM studies have described structural changes in the subbasal corneal nerve plexus and increased number of Langerhans cells (dendritic cells, DC) in patients with episodic or chronic migraine [12–17]. Corneal dendritic cells are bone marrow derived antigen-presenting cells and part of the corneal immune system, have a crucial role in both innate and adaptive immunity. They are located predominantly in the basal epithelium or in the sub-basal layer [18–20].

In case of a migraine attack a significant number of inflammatory molecules are released, sensitizing peripheral and central trigeminovascular neurons. One key mediator is CGRP (calcitonin gene related peptide). Its expression has been proved in trigeminal ganglion neurons [21] as well as in Langerhans cells [22]. CGRP inhibitors were the first class of drugs developed to prevent migraine.

The aim of our study was to evaluate corneal ultrastructural changes including the morphology of corneal subbasal nerve fibers and density and area of dendritic cells using in vivo confocal microscopy and to correlate it to tear film parameters applying a novel imaging tool (LacryDiag). We compared the data of the side primarily affected by migraine to contralateral data as well as to healthy controls.

## Patients and methods

All patients were referred to the Outpatient Headache Department of the Department of Neurology, Medical School, University of Pécs, Hungary between July 2022 and March 2023 and met the criteria of migraine as defined by the International Headache Society (3rd edition) [2]. All participants underwent a structured neurological examination (history taking, physical examination, blood pressure measurement, serum and urine tests, brain MRI study); migraine type, dominantly affected side, disease duration and attack frequency were determined. Patients were subdivided into 3 groups concerning disease duration (0–10 years, 11–20 years, |> 20 years). Similarly, 3 categories were defined regarding monthly attack frequency (rare: 0–5 attacks/month, average: 6–10 attacks/month, very frequent: 11–15 attacks/month). For the ophthalmological examination patients without major comorbidities, such as hypertension, cardiac disease, diabetes, thyroid gland dysfunction, oncological and hematological diseases, infectious diseases (e.g. HIV, hepatitis), central nervous system demyelination (e.g. multiple sclerosis), peripheral neuropathy and genetically inherited disorders (e.g. CADASIL) were selected. None of the patients suffered from menstrual migraine.

A comprehensive ophthalmologic examination including visual acuity, intraocular pressure, slit-lamp examination with fundus analysis, corneal tomography (Anterion; Heidelberg Engineering GmbH, Heidelberg, Germany), LacryDiag (Quantel Medical, France), and in vivo confocal microscopy (Heidelberg Retina Tomograph II Rostock Cornea Module; Heidelberg Engineering GmbH, Heidelberg, Germany) was performed in an interictal state. Patients with prior corneal or intraocular surgery and contact lens wear were excluded.

All study subjects underwent in vivo confocal microscopy of all corneal layers as described previously [23, 24],. Three good quality snapshots of the subbasal nerve plexus were selected in three different areas of the central cornea and they were analyzed with ACCMetrics software V3 (University of Manchester, Manchester, UK) [25–29]. Corneal nerve fiber density (NFD), the number of nerve fibers/mm^2^; nerve branch density (NBD), the number of primary branch points on the main nerve fibers/mm^2^; nerve fiber length (NFL), the total length of nerves mm/mm^2^; nerve fiber total branch density (TBD), the total number of branch points/mm^2^, nerve fiber area (NFA), the total nerve fiber area mm^2^/mm^2^; and nerve fiber width (NFW), the average nerve fiber width mm/mm^2^ and fractal dimension (FD) were evaluated.

To measure the dendritic cell (DC) area on IVCM images, Threshold Function of ImageJ software (http://imagej.nih.gov/ij/; National Institutes of Health, Bethesda, MD, USA) was utilized. The area of all DCs in three images for each subject were analyzed. Only mature DCs with branches (dendrites) were included in the cell count and cell area measurements.

All IVCM examinations were acquired by two experienced examiners (ZK, ES). The image selection and analysis for the IVCM were carefully reviewed by two independent examiners (AP, NT) and low-quality IVCM images or presence of any motion artifacts were excluded from the analysis.

Several tear film parameters (lower tear meniscus height measuring [LTMH], superior eyelid meibography [Meib], interferometry [INT], non-invasive break-up time [NIBUT]) were defined using LacryDiag (Quantel Medical, France), a novel non-invasive tear film imaging tool as described elsewhere [30]. The analyses was performed by an experienced examiner (NT). All migraine and control patients filled out the Ocular Surface Disease Index (OSDI) questionnaire.

The study was performed in accordance with the tenets of the Helsinki Declaration and the protocol was approved by the University of Pécs Institutional Ethical Review Board (Number: 9535-PTE 2023).

### Statistical analysis

Data were analyzed using the SPSS Statistics 25.0 (IBM Corp., Armonk, NY), MedCalc Version 14.8.1 (MedCalc Software, Ostend, Belgium) and Prism 9.4.1 for macOS (GraphPad Software, San Diego, CA, USA). For each data set, mean, standard deviation (SD) and 95% confidence interval (95% CI) for the mean were calculated. The non-parametric Mann–Whitney U test was carried out to compare data of controls to the side dominantly affected by the headache and with the unaffected side, analysis of variance (ANOVA) test was applied for subgroup analysis. For bivariate correlation analysis, the Spearman’s rank correlation “r” was used. Multiple logistic regression was applied to tease out the confounding effect of dry eye on corneal parameters. A P value below 0.05 was considered statistically significant.

## Results

87 eyes of 44 patients suffering from migraine (7 men and 37 women) with a mean age of 33.23 ± 11.41 years (range: 18 to 59 years) were recruited in our study and compared to 25 eyes of 25 healthy volunteers (6 men and 19 women) with a mean age of 30.16 ± 12.59 years (range: 22 to 79 years) (*P* = 0.190). All patients suffered from episodic migraine with an average disease duration of 16.02 ± 11.17 years and a monthly attack frequency of 4.37 ± 0.86. The dominantly affected side was the right side in 25 patients, the left side in 8 patients, and 11 patients showed bilateral involvement.

**Fig. 1:**
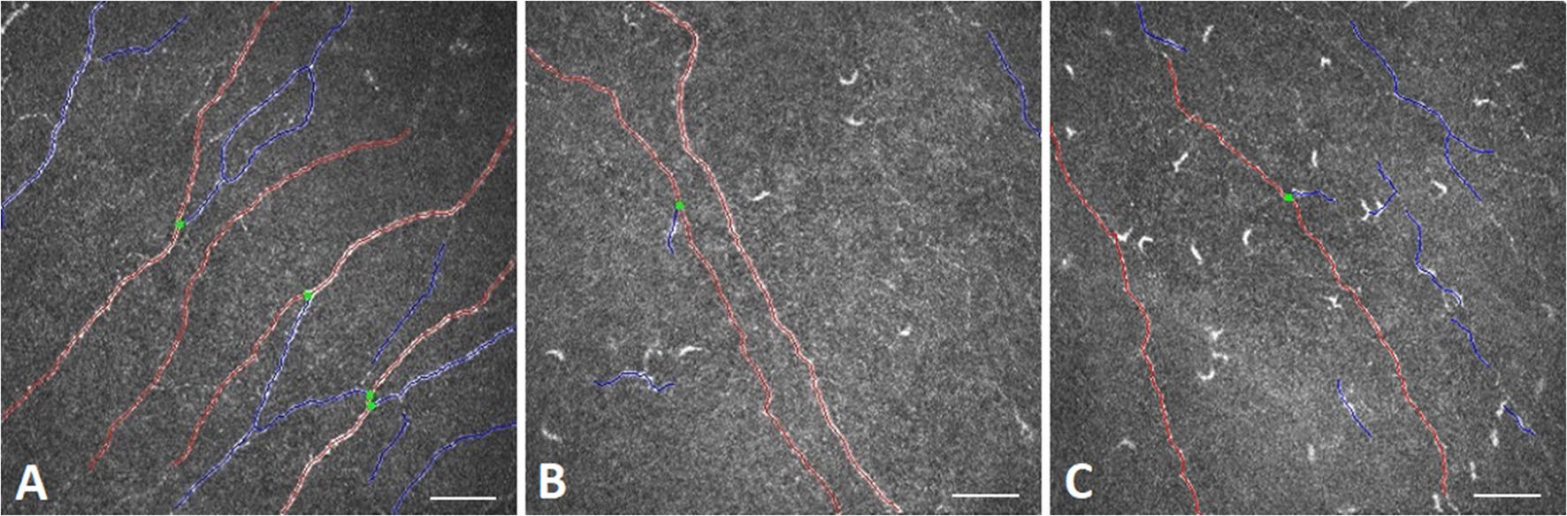
In vivo confocal microsocopy image of the corneal subbasal nerve plexus with annotation using the ACCMetrics software (red: nerve fiber, blue: nerve branch, green: branch point). **A:** Normal nerve fiber morphology of a 41-year-old healthy female; **B:** right eye (non-dominant side), **C:** left eye (dominant side) with decreased nerve fiber density, altered morphology and scattered dendritic cells of a 41-year-old patient with episodic migraine for 21 years with a monthly attack rate of 5. Scale bar represents 50 μm

Regarding the subbasal nerve fibers of the cornea, no significant difference could be recorded in NFD, NBD, TBD, and FD in the migraine group compared to controls (*P* > 0.05) (Table 1). However, we found a significant increase in the corneal DC density (*P* < 0.0001) and DC area (*P* < 0.0001) in migraine patients compared to healthy volunteers (Table 1, Fig. 1). Furthermore, the DC density showed a positive correlation with the monthly attack frequency (r = 0.307, *P* = 0.005) and DC area showed a negative correlation with NBD (r = -0.233, P = 0.039), NFL (r = -0.232, *P* = 0.040) and TBD (r = -0.233, *P* = 0.039).

**Table 1:** Subbasal nerve plexus morphology in patients with episodic migraine compared to healthy volunteers

	Healthy volunteers§	Patients with migraine§		P*
Nerve Fibre Density (No./mm^2^)	18.041 ± 7.107 (15.107 – 20.974)	16.827 ± 9.262 (14.041 – 19.985)		0.523
Nerve Branch Density (No./mm^2^*)*	19.186 ± 10.311 (14.930 – 23.442)	17.013 ± 13.694 (16.659 – 27.213)		0.233
Nerve Fibre Length (mm/mm^2^)	11.923 ± 3.105 (10.641 – 13.204)	11.965 ± 4.094 (11.077 – 12.854)		0.805
Nerve Fibre Total Branch Density (No./mm^2^)	33.581 ± 18.323 (26.018 – 41.144)	29.661 ± 18.667 (25.610 – 33.712)		0.326
Nerve Fibre Area (mm^2^/mm^2^)	0.005 ± 0.002 (0.004 – 0.006)	0.005 ± 0.002 (0.005 – 0.006)		0.455
Nerve Fibre Width (mm/mm^2^)	0.022 ± 0.002 (0.021 – 0.022)	0.022 ± 0.002 (0.021 – 0.022)		0.610
Fractal dimension	1.461 ± 0.036 (1.446 – 1.477)	1.458 ± 0.054 (1.446 – 1.470)		0.652
Dendritic cell density (cells/mm^2^)	24.64 ± 41.046 (7.697 – 41.583)	82.289 ± 60.633 (69.050 – 95.529)	< 0.0001	
Dendritic cell area (µm^2^)	34.161 ± 8.367 (30.707 – 37.615)	47.222 ± 9.538 (45.086 – 49.359)	< 0.0001	

^§^ Mean ± standard deviation (95% confidence interval)

^*^ Mann–Whitney U test

The Ocular Surface Disease Index (OSDI) did not show a significant difference between the control and migraine group (*P* = 0.499) (Table 2). Analyzing the tear film parameters with LacryDiag, a significantly higher loss of Meibomian gland area could be detected on the superior eyelid (*P* = 0.005) (Table 2) in migraine patients. All tear film parameters examined (LTMH, INT, NIBUT) proved not be significantly altered in migraine patients (Table 2).

**Table 2:** Tear film parameters in patients with episodic migraine compared to healthy volunteers

	Healthy volunteers§	Patients with migraine§	P*
Ocular Surface Disease Index	12.070 ± 7.139 (9.352 – 14.780)	12.570 ± 12.910 (9.175 – 15.960)	0.499
Lower tear meniscus height (mm)	0.220 ± 0.072 (0.191 – 0.250)	0.226 ± 0.103 (0.201 – 0.251)	0.764
Superior meibography (%)	6.087 ± 9.405 (2.020 – 10.150)	9.638 ± 5.327 (8.358 – 10.917)	0.005
Interferometry (score)	3.160 ± 1.214 (2.659 – 3.661)	3.667 ± 1.492 (3.316 – 4.017)	0.181
Non-invasive tear break-up time (sec)	10.288 ± 3.107 (8.976 – 11.599)	10.877 ± 3.957 (9.854 – 11.899)	0.224

^§^ Mean ± standard deviation (95% confidence interval)

^*^ Mann–Whitney U test

**Fig. 2:**
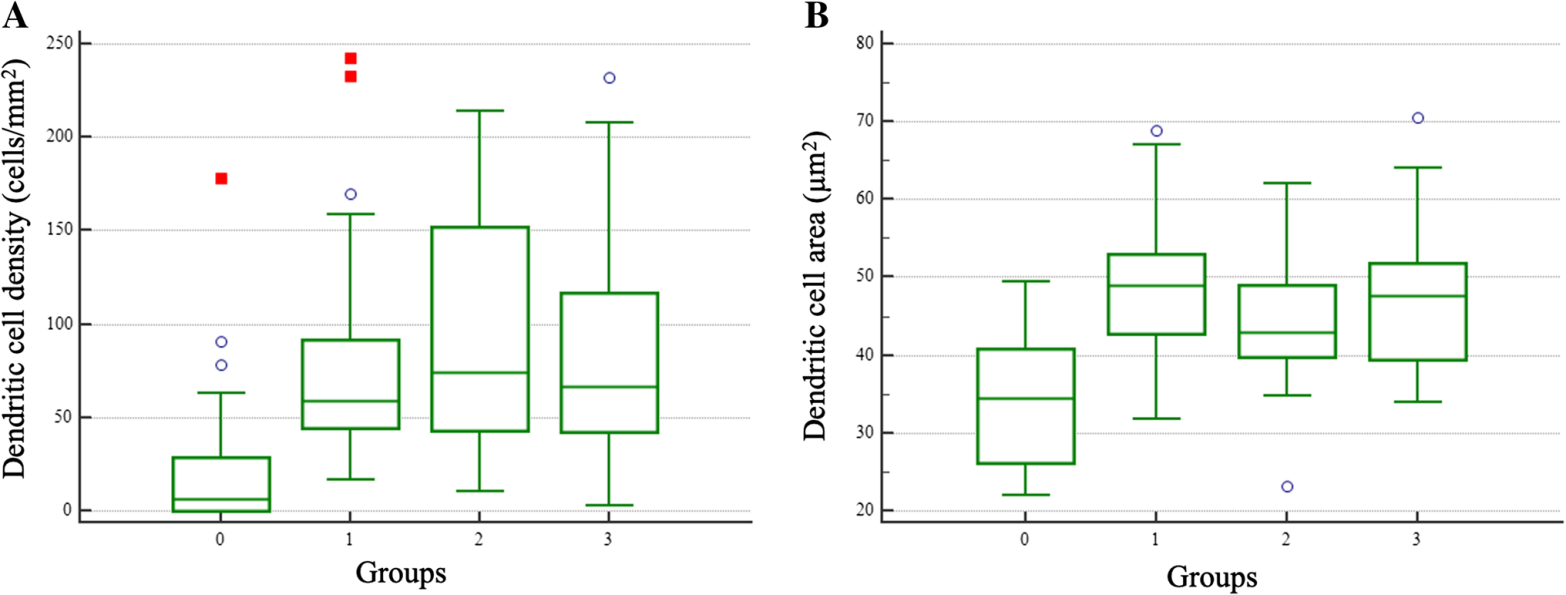
Box-and-whisker plot of parameters that showed significant difference (ANOVA, P < 0.001) in the subgroup analysis regarding disease duration (Group 0: healthy controls, Group 1: 0–10 years, Group 2: 10–20 years, Group 3: > 20 years of migraine duration). In all migraine subgroups a significant increase of **A:** DC density and **B:** DC area could be detected

In our subgroup analysis of disease duration, ANOVA showed a significant difference in dendritic cell density (*P* < 0.001) and dendritic cell area (*P* < 0.001) (Fig. 2). Post-hoc Tukey–Kramer test disclosed significant difference between the healthy group and each migraine group. Comparing the dominantly affected side to the contralateral side, none of the examined parameters showed a significant difference. Subgroup analysis by gender showed no significant differences regarding DCa, DCd and all nerve fiber parameters.

Teasing out the possible confounding effect of dry eye on the differences found in corneal parameters between the migraine and the control group using multiple linear regression; none of the covariables (OSDI, NIBUT) had a significant influence on the results obtained.

## Discussion

The trigeminal nerve plays a fundamental role in the function of the ocular surface unit [1]. It provides among others the sensory innervation to the cornea and conjunctiva. Above all, the cornea is particularly abundantly innervated by the trigeminal nerve, which regulates tear production, maintains corneal transparency, and protects the eye from injury. The trigeminal nerve also innervates the lacrimal gland regulating production and helps to maintain the integrity of the tear film. All components of the ocular surface functional unit are interconnected and work together to maintain the health and function of the ocular surface [1]. Disruption or dysfunction of any one component can lead to a range of ocular surface disorders, including dry eye syndrome.

In vivo corneal confocal microscopy, in particular, allows the detailed structural imaging of the cornea in health and disease. It has been well established previously, that IVCM shows excellent correlation with immunohistochemistry in determining the density of DCs in the human cornea [31]. Recent studies have described a significantly increased DC density under several medical conditions including dry eye disease [32],infectious keratitis [33], systemic autoimmune diseases [34, 35] and after SARS-CoV-2 infection [24]. A meta-analysis showed that mean DC density was 26.4 ± 13.6 cells/mm^2^ at the central cornea of healthy subjects [36] which corresponds well to our results. Compared to the controls DC density in migraine patients was more than 3 times elevated. A previous study has already described the aggregation of dendritic cells in close proximity of the corneal subbasal nerves in 10 migraine patients [12]. We could confirm this results in a larger cohort. In addition, we found significantly elevated DC area, suggesting the activation of these cells. Irrespective of the side of the headache, a general activation of a peripheral inflammatory process of the trigeminal system is indicated. Former studies have demonstrated that dendritic cells are involved in the modulation of nociception and pain through their effect on T cells [37]. The dendritic cell mediated inflammation of the trigeminal fibers might play a role in the positive feedback cycle of nociception and inflammation in migraine. As the DC area correlated with the monthly attack frequency, it would be worth to follow patients longitudinally or even monitor the changes of neuroinflammation in the cornea under a certain therapy. CGRP inhibitors are broadly used to prevent migraine. Calcitonin gene-related peptide levels have been shown to be elevated in the tear fluid of interictal migraine patients compared to healthy controls [38]. Knowing that dendritic cells express CGRP [22], their higher density and activated state correlates well with the previously described elevated CGRP levels in tear film.

Reduced corneal nerve fiber density and symptoms of dry eye have been published in chronic migraine patients [14]. In contrast, in episodic migraine patients the presence of nerve fiber regeneration has been suggested [22]. In our study, decreased nerve fiber density, branch density, total branch density and fractal dimension was observed in episodic migraine; however, the difference was not significant between the migraine and healthy group. Corneal dendritic cells and the trigeminal nerve are closely linked because the dendritic cells are in constant communication with the nerve fibers. The dendritic cells can activate the nerve fibers to trigger protective reflexes, and the nerve fibers can in turn modulate the activity of the dendritic cells. This bidirectional communication helps to ensure that the cornea is able to detect and respond to threats while maintaining its transparency and overall health. Disorders of the trigeminal nerve can have a significant impact on the function of the ocular surface. Damage to the ophthalmic branch of the trigeminal nerve can lead to decreased corneal sensitivity and tear production, which can result in dry eye syndrome and other ocular surface disorders. Several studies suggested an association of dry eye disease with migraine [39, 40]. CGRP found in the tear film is mainly generated by peptidergic sensory neurons of the cornea and conjunctiva, the lacrimal glands and meibomian glands have a minor contribution [41], furthermore CGRP is expressed by dendritic cells. The concentration of CGRP in the tear film of patients with dry eye disease is reduced [42], and the decrease in ocular surface sensitivity in patients with dry eye may account for it. In contrast, tear CGRP levels are significantly higher in interictal migraine patients compared to healthy controls [38]. Thus, the confounding effect of a dry eye disease should be taken into account when evaluating CGRP in the tear film of migraine patients; in particular, knowing the relevant comorbidity [43, 44].

The hypothesis that the dominantly affected side could show more dysfunctional nerve fibers and DCs emerged and has been investigated but could not be confirmed. The trigeminovascular theory of migraine describes a repetitive activation of this pathway leading to chronic changes in the central nervous system (CNS), migraine is regarded as a generalised brain disorder [10]. Correspondingly, Gunes et al. described a thinner retinal nerve fibre layer (RNFL) in patients with migraine compared to controls, but no statistically significant asymmetry in RNFL comparing the side dominantly affected by the headache with the non-dominant side [45].

Patients have been traditionally categorized as having a normal ocular surface (0–12 points) or as having mild (13–22 points), moderate (23–32 points), or severe (33–100 points) ocular surface disease based on the OSDI score [46]. In our study, healthy participants had an OSDI score of 12.070 and migraine patients had a score of 12.570. The comprehensive tear film imaging tool used in our study showed significantly greater loss of meibomian gland area in the superior eyelid in patients with episodic migraine. Altered meibography score can be characteristic of meibomian gland dysfunction [47]; however, no diagnostic cut-off has been established yet using LacryDiag. Though LacryDiag is validated and compared to other clinically used tear film analysing tools, it shows significant differences in meibography parameters between observers, likely influenced by the use of repeated testing and the non-dry eye cohort [48]. In our study one examiner (TN) carried out the analyses of LacryDiag data, in order to avoid the intergrader variability [30]. None of the other tear film parameters showed any alterations in migraine. A recent meta-analysis concluded that migraine headache was related to a higher risk of dry eye disease suggesting that headaches could be an independent risk factor for dry eye disease [49].

In conclusion, our results suggest the presence of neuroinflammation in the cornea of migraine patients affecting the peripheral trigeminal system. Dendritic cells surrounding the subbasal plexus may be involved in the activation and modulation of pain in migraine. To the best of our knowledge, this is the largest cohort of patients with episodic migraine examining the ocular surface functional unit. Timely recognition of changes in the peripheral nervous system reflected by corneal pathology is indispensable for understanding the pathogenesis of migraine and improving future therapies.
